# Another Case of Pulmonary Edema or May Be Not: An Unusual Presentation of Metastatic Melanoma

**DOI:** 10.14740/wjon734w

**Published:** 2014-08-25

**Authors:** Debabrata Bandyopadhyay, Praveen Vijhani, Carol Farver, Chirag Choudhary

**Affiliations:** a Pulmonary, Allergy and Critical Care Medicine, Respiratory Institute, Cleveland Clinic Foundation, Cleveland, OH, USA; b Department of Internal Medicine, Cleveland Clinic Foundation, Cleveland, OH, USA; c Department of Anatomic Pathology, Pathology and Laboratory Medicine Institute, Cleveland Clinic Foundation, Cleveland, OH, USA

**Keywords:** Acute respiratory distress, Metastatic melanoma, Lymphoma

## Abstract

Melanoma is a tumor of pigment producing cells melanocytes. Malignant melanoma is associated with a high morbidity and mortality because of its widespread and rapid metastasis. Melanoma commonly metastasizes to lung and secondary metastatic pulmonary melanoma is a well known entity. Metastatic melanoma can present with varied pattern of pulmonary involvement ranging from post obstructive pneumonia to atelectasis. However, lung involvement is not known to cause hypoxic respiratory failure. Here, we describe a rare case of metastatic melanoma presenting as an acute respiratory distress syndrome requiring mechanical ventilation.

## Introduction

Melanoma is a serious form of cancer commonly arising from skin melanocytes. Over 30% of patients with skin melanoma develop metastatic disease at the time of diagnosis, which usually entails a poor prognosis [1]. The median survival of metastatic melanoma is only 7.5 months [2]. The lung is one of the most common site of metastasis with 5% of all secondary lung malignancies due to melanoma [3]. Secondary melanoma of lung is very common along with distant lymph nodes metastasis, together accounting for 70-82% of all metastatic disease [4]. The prognosis of pulmonary metastatic melanoma is very poor with a 2-year survival of only 23% [5].

Metastatic melanoma to the lung is typically endobronchial and manifests clinically as symptoms of cough and hemoptysis with imaging studies revealing post obstructive pneumonia, lobar collapse or atelectasis [6]. In a series described by Chen and colleagues, the patterns of pulmonary involvement included solitary or multiple nodules, miliary pattern, mediastinal and/or hilar adenopathy and pleural effusion [7]. However, lung involvement is not known to cause hypoxic respiratory failure, specifically acute respiratory distress syndrome (ARDS) requiring mechanical ventilation. Although this situation is difficult to treat, an early diagnosis may be helpful in evaluating treatment options with newer immunotherapeutic agents thereby improving survival.

## Case Report

A 57-year-old ex-smoker Caucasian male with 20 pack-years of tobacco use was admitted with new onset and progressively worsening exertional dyspnea, orthopnea, and paroxysmal nocturnal dyspnea for the last 2 weeks accompanied with productive cough and yellow expectoration. He also developed bilateral leg swelling in the interim. Pertinent negative history includes fever, heartburn, chest pain, hemoptysis, any recent travel or exposure history. His past medical history was significant for coronary artery disease with three previous myocardial infarctions, diabetes mellitus and Hodgkin’s lymphoma 30 years ago. His lymphoma involved cervical and axillary lymph nodes without any associated B-symptoms. It was treated with chemotherapy and local radiotherapy. Five months prior, he developed a right-sided neck mass - a darkly pigmented discrete nodule of 3.5 mm diameter without any superficial ulceration. The excision biopsy showed S100 and HMB-45 positive melanoma cells, invasive to Clark level IV with breslow thickness of 2 mm. No tumor was present at surgical margins but sentinel lymph nodes were positive. Subsequently, he underwent radical neck dissection. He also received radiotherapy of 30 Gy in five fractions along with interferon alpha. His family history was negative for any malignant disorder.

On examination, he was afebrile, normotensive with sinus tachycardia, respiratory rate of 20 breaths/min, saturating 93% on room air. No jugular venous distension was noted but he had bilateral pedal edema on admission. Cardiovascular system examination revealed normal heart sounds, a pansystolic murmur at left Para-sternal area while chest auscultation revealed bibasilar fine crackles and decreased air entry at right lung base. The rest of his examination was unremarkable.

**Figure 1:**
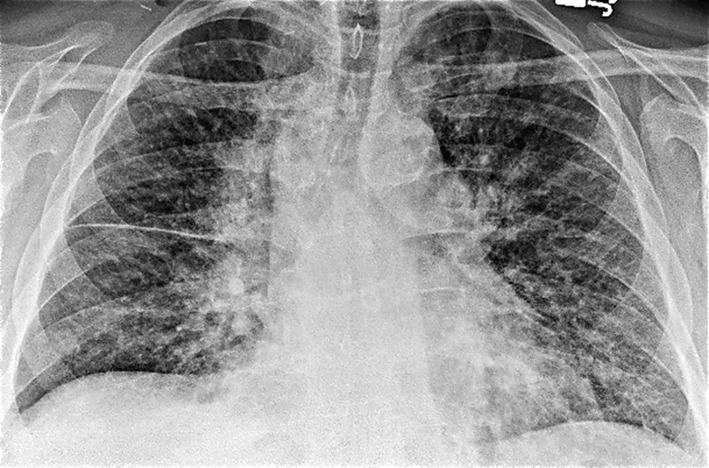
Chest X-ray on admission showed bilateral perihilar haziness and fluid in right transverse fissure consistent with pulmonary edema.

**Figure 2:**
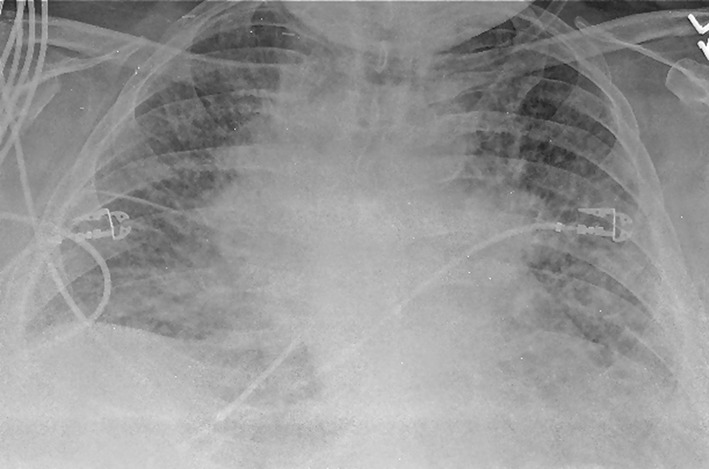
A repeat chest X-ray (day 7) revealed diffuse bilateral infiltrates or edema, predominantly interstitial in character with cardiomegaly (not significantly improved from admission, in spite of aggressive diuresis).

**Figure 3:**
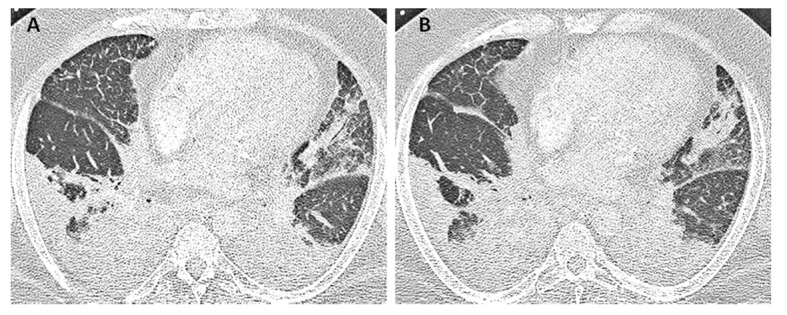
Patient’s CT scan of chest on day 7 (A, B) demonstrated mediastinal lymphadenopathy, diffuses pulmonary nodules, multifocal consolidations in right upper and lower lobe, lingular lobe, and left lower lobe as well as bilateral pleural effusion.

Laboratory studies were significant for anemia with hemoglobin at 11 (12 - 15.5 g/dL) and leukocytosis with WCC 15.41 (3 - 11 × 10^3^ mL). His creatinine was 0.67 (0.3 - 1.2 mg/dL) and B natriuretic peptide level was within normal limit at 53 (0 - 99 pg/mL). Chest X-ray showed bilateral perihilar haziness, consistent with pulmonary edema (Fig. 1). His electrocardiogram revealed sinus tachycardia with right bundle branch block. An echocardiogram demonstrated a good left and right ventricular systolic function (left ventricular ejection fraction 59%), grade-1 diastolic dysfunction with trivial tricuspid regurgitation. A diagnosis of diastolic heart failure was considered and patient was treated with diuretics, aiming daily negative fluid balance of 500 - 1,000 mL. He had lost 2 kg of weight since admission within a week with improvement of his pedal edema. However his breathing continued to deteriorate even after aggressive diuresis. On the seventh day of admission, he became tachypneic and hypoxic with arterial blood gas showing hypoxic and hypercarbic respiratory failure (pH 7.28, pO_2_ 78 mm Hg and pCO_2_ 76 mm Hg on FIO_2_ of 1.0). A repeat chest X-ray revealed diffuse bilateral infiltrates, predominantly interstitial in character, worsened compared to previous chest X-ray (Fig. 2). He required intubation and support with mechanical ventilation. Patients CT scan of chest (Fig. 3) demonstrated mediastinal lymphadenopathy, diffuse pulmonary nodules, multifocal infiltrates worse in right upper lobe, lower lobe, lingular lobe, and left lower lobe as well as bilateral pleural effusion.

**Figure 4:**
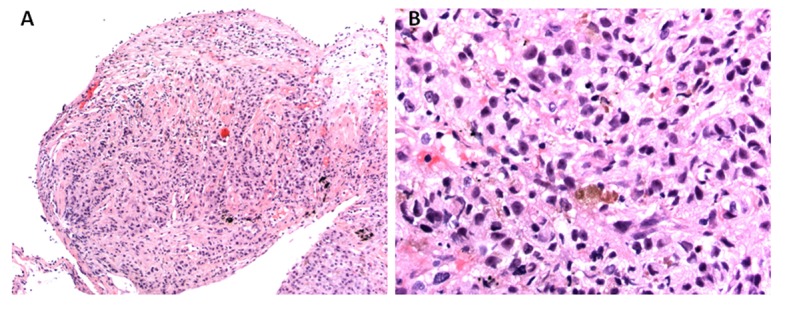
Histopathology of transbronchial biopsy from right upper lobe: (A) × 100 magnification and (B) × 400 magnification, showing malignant epithelioid cells with immunohistochemical staining positive for S100, Melan A and MITF, morphologically consistent with metastatic melanoma.

Given bilateral infiltrates and no significant cardiac dysfunction on echocardiogram, a diagnosis of ARDS was made. Moreover, his pO_2_/FIO_2_ ratio was less than 100 fulfilling Berlin criteria of severe ARDS definition [8]. An ARDS in the presence of lymphadenopathy, multiple pulmonary nodules as well as multifocal infiltrates conjure a broad differential. His bronchoscopy on eighth hospital day did not show any significant airway secretions or any mucosal lesions. Bronchoalveolar lavage revealed red blood cells of 76/mm^3^, white blood cell count of 850/mm^3^ neutrophil count 61%, lymphocytes 21%, monocytes 2%, and macrophages 6%. No malignant cells were seen. The bronchoalveolar lavage did not grow any virus, bacteria, fungus or mycobacteria. He received broad-spectrum antibiotics in form of vancomycin, azithromycin, pipercillin-tazobactam and micafungin without any significant improvement. Lymphoma has been uncommonly reported as causing ARDS but it is extremely unusual for melanoma to do so [9-11]. A lymphoproliferative disorder was excluded by flow cytometry of bronchoalveolar lavage. Subsequently on 12th day of admission, he underwent right upper lobe transbronchial biopsy (Fig. 4), which was positive for malignant epithelioid tumor, morphologically similar to malignant melanoma from his previous neck mass biopsy. Oncology was consulted for management of this patient’s melanoma. However, as per the oncologist’s opinion, no chemotherapy or biological agents could possibly improve his symptoms. Considering his very poor prognosis, the patient was transitioned to palliative care as per his family’s wish after 14 days of hospitalization.

## Discussion

ARDS is a form of acute lung injury characterized by diffuse damage to alveolar epithelium and pulmonary vascular endothelium. It is most commonly caused by infections, drugs and toxins, immunological disorders. Rarely malignant conditions have been reported as presenting as ARDS. This includes leukemia, lymphoma, choriocarcinoma, angiosarcoma, and adenocarcinona [11, 12]. Metastatic melanoma commonly involves lung as an endobronchial mass, solitary or multiple pulmonary nodules, hilar or mediastinal lymphadenopathy and pleural effusion [13]. The patchy infiltrative or ground glass metastatic pattern, mimicking pulmonary edema or pneumonitis is unusual. Chen and Dwyer, each reported two such cases [7, 14]. However diffuse infiltration of lung by melanoma presenting as ARDS has not been described. Metastatic lung melanoma is not known to cause hypoxia, respiratory failure or require mechanical ventilation. Shin et al described a case of reticulonodular pattern and ground glass opacities as well as pleural effusion in metastatic melanoma. The patient also had a high PA-Pa gradient but followed a sub-acute course and did not develop ARDS [15]. One patient described by Dwyer had diffuse pulmonary metastasis and presented in acute respiratory failure requiring ventilatory support following an open lung biopsy procedure [14].

It is postulated that melanoma metastasizes by gradual build up of tiny fractions of tumor cells leading to tumor nodules. The lung is at risk of abrupt embolic showers from these tumor nodules thereby causing multiple diffuse foci of metastasis. The resulting interstitial infiltration by embolic tumor cells leads to alveolar septal thickening which causes a defect in diffusion from the alveolus to the capillary, leading to increased PA-Pa gradient and hypoxemia [14, 15].

Around 160,000 new cases of melanoma are diagnosed each year worldwide and Caucasian population is at a 10 times greater risk of developing malignant melanoma [4]. The incidence of melanoma is increasing by 5-7% annually [5]. It is the most widely metastasizing neoplastic disease usually associated with a poor prognosis. Various chemotherapeutic agents have been used with reasonably good response, including dacarbazine and immunotherapy with interleukin-2 or recombinant interferon-alpha 2b. Newer drugs such as BRAF kinase inhibitor (vemurafenib) have been shown to produce progression-free survival. Other agents such as gp100 peptide vaccine and ipilimumab, an antibody to cytotoxic T lymphocyte antigen, are promising treatment options in the area of metastatic disease [16].

This case demonstrates an important lesson we must consider - the diagnostic possibility of metastatic malignant melanoma in an appropriate background in a patient with ARDS. A high degree of suspicion should be harbored when assessing these groups of patients, as an early diagnosis may be helpful in evaluating effective newer treatment modalities, thus prolonging survival.

## References

[1] Essner R, Lee JH, Wanek LA, Itakura H, Morton DL (2004). Contemporary surgical treatment of advanced-stage melanoma. Arch Surg.

[2] Barth A, Wanek LA, Morton DL (1995). Prognostic factors in 1,521 melanoma patients with distant metastases. J Am Coll Surg.

[3] Mohan KM, Gowrinath K (2010). Unusual thoracic manifestation of metastatic malignant melanoma. Lung India.

[4] Lee YT (1980). Malignant melanoma: pattern of metastasis. CA Cancer J Clin.

[5] Balch CM, Gershenwald JE, Soong SJ, Thompson JF, Atkins MB, Byrd DR, Buzaid AC (2009). Final version of 2009 AJCC melanoma staging and classification. J Clin Oncol.

[6] Briones Gomez A, Cases Viedma E, Domenech Clar R, Sanchis Aldas JL (1999). [Pulmonary metastases of malignant melanoma. A rare endobronchial presentation]. Arch Bronconeumol.

[7] Chen JT, Dahmash NS, Ravin CE, Heaston DK, Putman CE, Seigler HF, Reed JC (1981). Metastatic melanoma in the thorax: report of 130 patients. AJR Am J Roentgenol.

[8] Ranieri VM, Rubenfeld GD, Thompson BT, Ferguson ND, Caldwell E, Fan E, Camporota L (2012). Acute respiratory distress syndrome: the Berlin Definition. JAMA.

[9] Wood SM, Boyd SM, Taylor JE, Savill J (1996). A case of non-Hodgkin lymphoma presenting primarily with renal failure. Nephrol Dial Transplant.

[10] Sahebjani H, Vassallo CL (1975). Rapidly progressive lymphoma of lung appearing as a adult respiratory distress syndrome. Chest.

[11] Kishimoto M, Prasertsuntarasai T, Gelber R, Tanabe A, Gallacher TS (2004). Diffuse large B-cell lymphoma manifesting as acute respiratory distress syndrome. Am J Med Sci.

[12] McGowan MP, Pratter MR, Nash G (1990). Primary testicular choriocarcinoma with pulmonary metastases presenting as ARDS. Chest.

[13] Loewenthal B, Shiau MC, Garcia R (2004). Metastatic melanoma: an unusual diagnosis for a large anterior mediastinal mass. Radiographics.

[14] Shin NY, Hong YJ, Kim AH, Shim HS, Nam JE, Lee HJ, Kim MJ (2011). Diffuse interstitial infiltrative lung metastasis of malignant melanoma: a case report. Korean J Radiol.

[15] Dwyer AJ, Reichert CM, Woltering EA, Flye MW (1984). Diffuse pulmonary metastasis in melanoma: radiographic-pathologic correlation. AJR Am J Roentgenol.

[16] Salama AK (2013). Evolving pharmacotherapies for the treatment of metastatic melanoma. Clin Med Insights Oncol.

